# Variability of indication criteria in knee and hip replacement: an observational study

**DOI:** 10.1186/1471-2474-11-249

**Published:** 2010-10-26

**Authors:** Raquel Cobos, Amaia Latorre, Felipe Aizpuru, Jose I Guenaga, Cristina Sarasqueta, Antonio Escobar, Lidia García, Carmen Herrera-Espiñeira

**Affiliations:** 1 Health Research Unit of Alava, Basque Health Service, José Achótegui s/n, 01009, Vitoria-Gasteiz, Álava, Spain; 2 Santiago Hospital, Olaguibel, 29, 01004, Vitoria-Gasteiz, Álava, Spain; 3 Donostia Hospital, Paseo Dr. Beguiristain s/n, 01009, San Sebastian, Guipuzcoa, Spain; 4 Basurto Hospital, Avda. Montevideo, 18, 48013, Bilbao, Vizcaya, Spain; 5 Canary Islands Foundation for Health Research (FUNCIS), Pérez de Rozas, 5, 4th Floor, 38004, Santa Cruz de Tenerife, Canary Islands, Spain; 6 Virgen de las Nieves Hospital, Avda. De Las Fuerzas Armadas, 2, 18014, Granada, Spain; 7 Network Center for Biomedical Research in Epidemiology and Public Health (CIBERESP), Spain; 8 University of Basque Country (UPV/EHU), Spain

## Abstract

**Background:**

Total knee (TKR) and hip (THR) replacement (arthroplasty) are effective surgical procedures that relieve pain, improve patients' quality of life and increase functional capacity. Studies on variations in medical practice usually place the indications for performing these procedures to be highly variable, because surgeons appear to follow different criteria when recommending surgery in patients with different severity levels. We therefore proposed a study to evaluate inter-hospital variability in arthroplasty indication.

**Methods:**

The pre-surgical condition of 1603 patients included was compared by their personal characteristics, clinical situation and self-perceived health status. Patients were asked to complete two health-related quality of life questionnaires: the generic SF-12 (Short Form) and the specific WOMAC (Western Ontario and Mcmaster Universities) scale. The type of patient undergoing primary arthroplasty was similar in the 15 different hospitals evaluated.

The variability in baseline WOMAC score between hospitals in THR and TKR indication was described by range, mean and standard deviation (SD), mean and standard deviation weighted by the number of procedures at each hospital, high/low ratio or extremal quotient (EQ_5-95_), variation coefficient (CV_5-95_) and weighted variation coefficient (WCV_5-95_) for 5-95 percentile range. The variability in subjective and objective signs was evaluated using median, range and WCV_5-95_. The appropriateness of the procedures performed was calculated using a specific threshold proposed by Quintana et al for assessing pain and functional capacity.

**Results:**

The variability expressed as WCV_5-95 _was very low, between 0.05 and 0.11 for all three dimensions on WOMAC scale for both types of procedure in all participating hospitals. The variability in the physical and mental SF-12 components was very low for both types of procedure (0.08 and 0.07 for hip and 0.03 and 0.07 for knee surgery patients). However, a moderate-high variability was detected in subjective-objective signs. Among all the surgeries performed, approximately a quarter of them could be considered to be inappropriate.

**Conclusions:**

A greater inter-hospital variability was observed for objective than for subjective signs for both procedures, suggesting that the differences in clinical criteria followed by surgeons when indicating arthroplasty are the main responsible factors for the variation in surgery rates.

## Background

Total knee replacement (TKR) and total hip replacement (THR) are safe surgical procedures that achieve effective pain reduction and adequate restoration of function in the vast majority of patients suffering from advanced knee, hip and other forms of osteoarthritis [1,2]. Furthermore, it is widely accepted that the functional benefits of these kinds of procedures exceed their clinical risks and costs [3].

The main reason for indicating these procedures is osteoarthritis, which in Spain affects around 10% of the general population [4]. Several studies have reported that the prevalence of osteoarthritis is high in the over 60's age group, ranging from 5 - 7.4% and 10.2-12.2% for hip and knee disease, respectively [4,5], although the method used to calculate the prevalence influences these values [6]. According to these authors, the disease is more common in women and tends to increase with age. Obesity is a risk factor for osteoarthritis, both for onset and progression, especially in the case of knee diseases; the relationship in hip osteoarthritis is much weaker [7,8].

Osteoarthritis is the most common form of arthritis. The condition is characterised by loss or failure of the functional and/or biochemical integrity of the joint. Osteoarthritis symptoms include joint pain, stiffness, and dysfunction, but the principal problem for the majority of patients is the pain [9]. Diseases of the musculoskeletal system have a clearly detrimental effect on health related quality of life [4,10] as they significantly affect basic activities such as climbing up or going down stairs and changing position from sitting to standing, as well as ambulating and mobility in general [11,12].

Tornero et al. reported that osteoarthritis is by far the main cause of rheumatic disease-related temporary and permanent disability and is the second cause of permanent disability after cardiovascular diseases [13]. It is also the main cause of pain and disability in the elderly and the main reason for hip- and knee-joint replacement [14].

In the medical literature reviewed there seems to be a consensus regarding the importance of pain and limited functional capacity when indicating surgical intervention [15-19], although there is some disagreement regarding the importance of other patient characteristics such as obesity, age, comorbidity, employment situation, range of active flexion and extension, pain when walking, feeling of instability, radiographic evidence of moderate or severe osteoarthritis, pain at night, and patient request for treatment, among others [19].

In the last years there has been a notable increase in the number of hip and knee arthroplasties. This increase, together with differences in intervention rates and clinical criteria [20], means that an analysis of the appropriate use of these procedures is required as inappropriate surgery leads to longer waiting lists and unnecessary increases in health expenditure.

Area variation in the rates of knee replacement may occur for many reasons, including differences in disease prevalence or severity, differences in patients' and surgeons' expectations and preferences for treatment, as well as restricted access to procedure. So, geographic variation in the rates not accounted for by disease prevalence or severity may represent underuse of the procedure in some areas, overuse in other areas or a combination of the two [19].

Variations in orthopaedic and musculoskeletal surgery have been reported in different countries such as the United States [21], Canada [22] and Spain, where a study undertaken in 2002 involving 111 health regions from 9 Autonomous Communities reported intervention rates that were five- (hip) and six-times (knee) higher in some centres than others [23]. This variability depends, amongst other factors, on the clinical criteria followed by surgeons in the different regions to prescribe the procedure for cases of different severity, meaning that a percentage of surgeries performed might be considered to be "inappropriate or unfounded" [24].

In order to determine the inter-hospital variability in the baseline WOMAC and clinical situation when indicating TKR or THR, a prospective multicentre study involving 15 Spanish hospitals from the Basque Country, Andalucia and Canary Islands was proposed. As the secondary objective, the number of inappropriate procedures performed was calculated on the basis of the minimal pain and functional capacity threshold proposed by Quintana et al [24].

## Methods

### Study design and inclusion/exclusion criteria

A multicentre prospective observational study in 15 hospitals from the Basque Country, Canary Islands and Andalucia involving patients over 18 years of age, diagnosed with primary knee or hip osteoarthritis who underwent total knee (CIE-9-MC: 8154) or total hip replacement (CIE-9-MC: 8151). Patients were selected from the waiting list databases for these surgical interventions in the participating hospitals between July 2005 and December 2006. Patients who were illiterate or suffering from a neuropsychiatric disorder were excluded.

This study was approved by the Health Human Research Ethics Committee of each participating hospital according to the Declaration of Helsinki. All patients gave informed consent to participate in the study.

### Measurements

In all the participating hospitals, the preoperative situation of patients was assessed on the basis of personal characteristics, clinical situation and self-perceived health status. Two health-related quality of life questionnaires were used: the WOMAC, which is disease specific, and a generic questionnaire (SF-12), both translated into Spanish and validated. The WOMAC questionnaire [25,26] evaluates three dimensions: pain, stiffness and functional capacity. Each dimension contains 5, 2 and 17 items respectively, all of which are scored on a Likert-type scale with 5 options of answer. Questionnaires with one blank item in the pain and stiffness dimensions and between one and three blank items in the functional capacity dimension were corrected according to the procedure proposed by Bellamy et al, with missing data for the unanswered items being replaced by the mean value of items answered for each dimension [27]. Questionnaires with two or more blank items in the pain dimension, two blank items in the stiffness dimension and four or more blank items in the functional capacity dimension were not considered in the statistical analysis in this section.

In order to compare the study outcomes with those reported in the literature, the score for each WOMAC dimension was standardized to a scale of 0 to 100 (where 0 represents the best possible health status and 100 the worst).

To assess the study's secondary objective, a procedure was considered to be inappropriate when WOMAC pain or functional capacity dimension scores were 40 or lower [24].

Version 1 of the SF-12 questionnaire [28], a shorter form of the SF-36, was used. This questionnaire includes 12 items that provide two summary scores, one each for the physical (Physical Health Composite Score -PCS-) and mental (Mental Health Composite Score -MCS-) components. These summaries range from 0 to 100 (where 0 represents the worst possible status -physical or mental- and 100 the best).

Both questionnaires were given to patients when they attended the hospital either to be scheduled for surgery or for blood extraction if they were included in a self-blood transfusion programme. They were asked to complete these questionnaires and return them upon admission to hospital.

The number of chronic diseases in addition to osteoarthritis and different subjective and objective signs appropriate to each process were considered to assess the clinical situation upon admission. In knee osteoarthritis, signs measured included pain while walking, pain at rest, ambulating capacity, stair climbing, getting up from a chair, limping, need for walking aids/wheelchair, muscle strength, active extension, passive extension, passive flexion and instability. For the hip osteoarthritis score, the subjective and objective signs assessed were pain at rest, pain while walking, ambulating capacity, limping, stair climbing, needed for walking aids/wheelchair, range of motion (abduction, adduction, internal rotation, external rotation, flexion and extension), Trendelenburg test and dysmetria. All signs were scored on different scales with between two and five answer options and subsequently grouped into two classes of severity: mild or moderate/severe. The values of these variables after reclassification are listed in Table 1.

**Table 1:** Subjective and objective signs by category

*SIGNS*	*JOINT*	*VARIABLES*	*STATUS*	
			*Mild*	*Moderate/Severe*
SUBJECTIVE SIGNS	Knee/Hip	Pain at rest	None Mild/occasional	Moderate Severe
		Pain while walking	None Mild/occasional	Moderate Severe
		Ambulating capacity	More than 100 meters and more than 15 minutes	Less than 100 meters and/or standing for less than 15 minutes. Cannot walk or stand
		Stairs climbing	Normal	With support of the banisters
		Limping	No limping Mild limping	Moderate limping Severe limping Wheelchair Confined to bed
	Knee	Getting up from a chair	Normal	With help
	Knee/Hip	Need for walking aids/wheelchair	None Stick or crutches needs just for long distances	Most of the time, one or two crutches Zimmer frame Wheelchair
OBJECTIVE SIGNS	Knee	Muscle strength	Counteracts gravity but does not counteract resistance Counteracts gravity and resistance	Slight muscular contraction Potential muscular contraction but insufficient to counteract gravity
		Active extension	> 5°	≤ 5°
		Degree of instability	≤ 5°	> 5°
		Passive extension	> 10°	≤ 10°
		Passive flexion	> 100°	≤ 100°
	Hip	Flexion	> 60°	≤ 60°
		Abduction	> 10°	≤ 10°
		Adduction	> 10°	≤ 10°
		Extension	> 10°	≤ 10°
		Internal rotation	> 10°	≤ 10°
		External rotation	> 10°	≤ 10°
		Trendelenburg	No	Yes
		Dysmetria	No	Yes

### Statistical analysis

The data were analysed using the SPSS (Statistical Package for the Social Sciences) software (Version 16.0).

Sample characteristics were described on the basis of the mean and standard deviation or the percentage distribution and 95% confidence interval (95% CI) depending on the continuous or categorical nature of the variables.

To describe the inter-hospital variability in hip and knee replacement indication, the range, mean and standard deviation (SD), mean and standard deviation weighted for the number of procedures undertaken in each hospital, extremal quotient (EQ_5-95_), coefficient of variation (CV_5-95_) and the weighted coefficient of variation for the 5-95 percentile range (WCV_5-95_) were calculated for pain, stiffness and functional-capacity dimensions on the WOMAC scale and for the physical and mental components of the SF-12 scale. The variability in subjective and objective signs was described by the median, range and WCV_5-95_.

The variability was considered low for values of CV_5-95 _or WCV_5-95 _of up to 0.30 and moderate to high for values of 0.30 or above [29,30].

The odds ratio (OR) was calculated to estimate the risk of undergoing an inappropriate intervention as a function of gender.

## Results

### Sample description

Among the 622 patients underwent THR, 336 were men (54.0%). Considering both sexes, in THR group, the mean (SD) age was 65.4 (12.2) years and the mean (SD) body mass index (BMI) was 28.2 (4.1), The mean number of chronic diseases in addition to osteoarthritis upon admission to hospital was 1.1 (1.1). Overall, 160 (47.6%) men and 88 (30.8%) women underwent THR before 65 years of age (p < 0.001).

Among the 981 patients underwent TKR, 698 were women (71.2%). The mean age of all the patients was 70.5 (7.4) and the mean BMI was 30.6 (4.8). The number of chronic diseases in addition to osteoarthritis upon admission to hospital was 1.5 (1.2). Overall, 66 (23.3%) men and 124 (17.8%) women underwent TKR before 65 years of age (p = 0.046).

### Patient Outcomes

The weighted mean of the WOMAC score for the THR group was 56.5 (4.9) for pain, 58.9 (6.2) for stiffness and 66.1 (4.2) for functional capacity, and the mean values for the physical and mental components of SF-12 questionnaire were 29.6 (2.6) and 42.7 (3.4) respectively.

The corresponding values for the TKR group were 57.0 (4.0) (pain), 58.7 (6.7) (stiffness) and 63.5 (4.6) (functional capacity) on the WOMAC scale and 29.8 (1.8) (physical component) and 42.2 (2.9) (mental component) on the SF-12.

The inter-hospital variability, expressed as WCV_5-95_, was very low (0.03-0.11) in all cases (Tables 2 and 3).

**Table 2:** Variability in the WOMAC questionnaire by intervention type

*VARIABILITY INDICATORS*	*WOMAC QUESTIONNAIRE*					
	*Pain*		*Stiffness*		*Functional capacity*	
	Hip	Knee	Hip	Knee	Hip	Knee
No. of cases	622	981	622	981	622	981
Range	47.5-68.3	51.7-65.5	45.1-67.2	45.7-67.3	61.1-82.5	53.3-72.5
Mean (SD)	57.1 (5.7)	57.2 (4.3)	59.4 (5.3)	59.5 (6.5)	67.4 (5.9)	63.8 (5.3)
Weighted mean (SD)	56.5 (4.9)	57.0 (4.0)	58.9 (6.2)	58.7 (6.7)	66.1 (4.2)	63.5 (4.6)
EQ_5-95_	1.4	1.24	1.5	1.47	1.3	1.32
CV_5-95_	0.08	0.06	0.08	0.11	0.06	0.06
WCV_5-95_	0.05	0.06	0.10	0.11	0.06	0.06

SD - Standard deviation

EQ_5-95 _- Extremal quotient for the 5-95 percentile range

CV_5-95 _- Coefficient of variance for the 5-95 percentile range

WCV_5-95 _- Weighted coefficient of variance for the 5-95 percentile range

**Table 3:** Variability in the SF-12 questionnaire by intervention type

*VARIABILITY INDICATORS*	*SF-12 QUESTIONNAIRE*			
	*Physical component*		*Mental component*	
	Hip	Knee	Hip	Knee
No. of cases	503	815	503	815
Range	25.1 - 36.0	25.5 - 33.8	31.5 - 45.9	35.9 - 49.4
Mean (SD)	29.7 (3.1)	29.5 (1.9)	41.9 (4.3)	42.0 (3.7)
Weighted mean (SD)	29.6 (2.6)	29.8 (1.8)	42.7 (3.4)	42.2 (2.9)
EQ_5-95_	1.4	1.3	1.5	1.3
CV_5-95_	0.08	0.05	0.08	0.08
WCV_5-95_	0.08	0.03	0.07	0.07

SD - Standard deviation

EQ_5-95 _- Extremal quotient for the 5-95 percentile range

CV_5-95 _- Coefficient of variance for the 5-95 percentile range

WCV_5-95 _- Weighted coefficient of variance for the 5-95 percentile range

### Subjective and objective signs of severity

In THR group, the objective signs with the greatest inter-hospital variation were "flexion ≤ 60°" (WCV_5-95 _= 0.62), "positive Trendelenburg" (WCV_5-95 _= 0.76) and "dysmetria" (WCV_5-95 _= 0.58), and the subjective signs were "ambulation limited to less than 100 meters and/or 15 minutes" (WCV_5-95 _= 0.50), "moderate to severe limp" (WCV_5-95 _= 0.34) and "need for walking aids - one or two crutches most of the time/Zimmer frame - or wheelchair" (WCV_5-95 _= 0.31). The results of the WOMAC variability by intervention type are reported in Table 4.

**Table 4:** Variability in subjective and objective signs by intervention type

*SIGNS*		*HIP*		*KNEE*	
		*Median (Range)*	***WCV***_***5-95***_	*Median (Range)*	***WCV***_***5-95***_
SUBJECTIVE SIGNS	Pain whilst walking (%)	93.2 (82.1-100)	0.06	94.1 (52.9-100)	0.12
	Pain at rest (%)	50 (7.1-72.7)	0.23	43.5 (7.7-80.5)	0.40
	Ambulating capacity (%)	26.9 (0.0-61.8)	0.50	27.3 (2.1-100)	0.42
	Stair climbing (%)	90.9 (69.2-100)	0.10	95.2 (63.8-100)	0.08
	Limping (%)	81.8 (26.1-100)	0.34	68.3 (19.1-100)	0.30
	Need for walking aids/wheelchair (%)	42.9 (10.0-80.0)	0.31	36.4 (8.3-100)	0.52
	Getting up from a chair (%)			64.7 (18.2-100)	0.23
OBJECTIVE SIGNS	Abduction (≤ 10°) (%)	36.1 (0.0-60.0)	0.38		
	Adduction (≤ 10°) (%)	60.0 (0.0-100)	0.43		
	Flexion (≤ 60°) (%)	18.2 (0.0-75.0)	0.62		
	Extension (≤ 10°) (%)	92.3 (36.2-100)	0.08		
	Internal rotation (≤ 10°) (%)	85.7 (44.8-100)	0.13		
	External rotation (≤ 10°) (%)	62.5 (19.3-92.3)	0.15		
	Trendelenburg (yes) (%)	3.8 (0.0-100)	0.76		
	Dysmetria (yes) (%)	33.3 (0.0-100)	0.58		
	Muscle strength (contraction not counteracting gravity)			9.1 (0.0-45.5)	0.52
	Active extension (≤ 5°) (%)			77.2 (30.0-100)	0.19
	Passive extension (≤ 10°) (%)			92.5 (50.0-100)	0.11
	Passive flexion (< 100°) (%)			41.7 (21.3-100)	0.34
	Instability degree (> 5°) (%)			20.5 (0.0-100)	0.69

WCV_5-95 _- Weighted variance coefficient for the percentile range 5-95

In the TKR group, the inter-hospital variability for objective signs was very high for "instability > 5°" and "muscle strength - contraction not counteracting gravity" with WCV_5-95 _values of 0.69 and 0.52, respectively. The subjective signs with greatest inter-hospital variability were "pain at rest", "ambulation limited to less than 100 meters and/or 15 minutes" and "need for walking aids - one or two crutches most of the time/Zimmer frame - or wheelchair", with WCV_5-95 _scores of 0.40, 0.42 and 0.52, respectively.

### Inappropriate interventions

Among all the surgeries performed, 153 hip operations (24.6%; 95% CI: 21.2%-28.0%) and 250 knee operations (25.5%; 95% CI: 22.8%-28.2%) were inappropriate according to the criteria used in this study. The range between hospitals varied between 10% and 35.7% for hip operations and between 7.3% and 41.7% for knee surgery. Patients who underwent an inappropriate TKR were older (72.0 (6.9) versus 69.9 (7.5); p < 0.001) and more likely to be men [33.2% (n = 94) versus 22.3% (n = 156), p < 0.001; OR 1.7 (95% CI: 1.3-2.3)] than members of the appropriate surgery group. Among the patients who underwent THR there were no significant differences between groups (appropriate or inappropriate intervention) in terms of age, but the same pattern was observed regarding gender [28.3% (n = 95) of men' interventions versus 20.3% (n = 58) of women' interventions were labelled as inappropriate, p = 0.022; OR 1.6 (95% CI: 1.1-2.3)].

Within the appropriate surgery group, men were younger than women for both types of procedures (mean age of men was 68.8 (7.8-standard deviation-) versus 70.3 (7.3) for women, p = 0.014 in TKR; and 63.0 (12.9) versus 68.0 (11.3), p < 0.001 respectively in THR), while in the inappropriate surgery group there was no significant difference between the mean age of men and women (p = 0.942 and p = 0.183 in TKR and THR respectively).

## Discussion

This study involved 981 patients who underwent knee arthroplasty and 622, hip arthroplasty. Both groups were similar in terms of age and gender distribution to those studied by other authors [24,31,32], with more women in the TKR group and a higher proportion of men undergoing hip surgery. In addition, patients of the TKR were older (70.5 (7.4) years) than those in the THR group (65.4 (12.2)).

Approximately a third of the women and half of the men in the THR group were younger than 65 years of age when they have been operated (p < 0.001). Previous studies [33,34] have suggested that osteoarthritis is more common in women than in men but that women tend to undergo arthroplasty at a more advanced stage of the disease. The mechanisms underlying these gender-related differences are not yet clear but may be related to the patient's level of activity, the duration of their symptoms and their level of disability. A study published by Karlson et al. reported that men had a higher level of activity and carried out heavier works than women. The study also found that men had greater confidence in the surgeon's ability and were less afraid of a negative surgical outcome than women. In contrast, women were more worried than men about being a burden on other people after the surgery and stated three reasons for postponing surgery: 1) they preferred to wait until they reached a certain level of pain or suffered loss of functional capacity or interference with an essential activity; 2) they were waiting for the technology to improve; and 3) the procedure could not be delayed any longer [34].

A similar difference was observed in the TKR group, with around a quarter of men and a fifth of women being operated before 65 years of age (p = 0.161).

The mean number of chronic diseases in addition to osteoarthritis upon admission was 1.1 (1.1) for the THR group and 1.5 (1.2) for the TKR group, possibly due to the higher average age of the patients undergoing knee surgery. These values are similar to rates of chronic diseases in the general population in Spain [1.4 for patients between 65 and 69 years of age and 1.6 and for those between 70 and 74 years of age [35]].

In the THR group 449 patients (80.5%) and in the TKR group 752 patients (89.1%) were overweight or obese (BMI ≥ 25) (p < 0.001). These values are similar to those reported previously (8, 36) and confirm that obesity is a more important risk factor for knee osteoarthritis than for hip osteoarthritis.

The scores obtained on both components of the SF-12 were lower (worse) than those of the general population [28], and similar to results from other researchs on THR and TKR, such as that reported by Hamel et al [37]. In both cases, the variation in osteoarthritis-specific (WOMAC) and in the general (SF-12) questionnaires between the 15 participating hospitals was very small (WCV_5-95 _between 0.03 and 0.11).

There is general agreement among physicians that pain should be considered the principal criterion for determining who is a candidate for primary hip or knee arthroplasty [15,38]. In line with this, the percentage of patients undergoing THR with severe "pain when walking" was over 90% in half of the hospitals we studied (82.1% in the hospital with the lowest rate). In the TKR group, the percentage of patients reporting severe pain when walking before the surgery was also above 90% in half of the hospitals, but the figure for one of the hospitals was as low as 52.9%. Nevertheless, the overall variability was small (0.06-0.12) in both cases.

In contrast, the percentage of patients with severe "pain at rest" prior to intervention was less than 50% and 43.5% for THR and TKR respectively in the half of the hospitals. Moreover, the inter-hospital variability in this case was low (WCV_5-95 _= 0.23) in the THR group but moderate to high (WCV_5-95 _= 0.40) in the TKR group. In both cases, there were hospitals with very low percentages of patients (7.1%; 1 of 14 in hip arthroplasty) and (7.7%; 1 of 13 in knee arthroplasty), suffering from severe pain at rest. This variability in the objective signs might reflect different cultural perceptions of pain according to geographical origin. It might also suggest the difference among clinicians when assesing pain, since these signs were obtained by physicians upon admission.

Looking at the other subjective signs assessed, except for "the need to hold on to the banister when climbing stairs" in both types of interventions and for "getting up from a chair" in the TKR group, the variability was moderate - for "limping" (WCV_5-95 _= 0.34 in THR and 0.30 in TKR) and "need for walking aids/wheelchair" in the THR group (WCV_5-95 _= 0.31), or moderate to severe (WCV_5-95 _≥ 0.40) for "limitations in ambulating" in both types of interventions and for "need for walking aids/wheelchair" in the TKR group. Once again, this variation highlights the fact that physicians follow different clinical criteria when indicating surgery within the same country, i.e., in similar populations suffering from the same diseases.

On the other hand, the large variability observed in the objective signs analysed could be due to inadequate data collection.

In a study published in September 2009, Quintana et al. proposed minimum scores on the WOMAC pain and functional capacity scales above which surgery would be indicated to achieve a clinically relevant improvement of 30 and 25 points on the pain and functional-capacity scales, respectively. This gain was clinically relevant for pre-intervention WOMAC functional limitation domain values > 60 or between 40 and 60 accompanied by a level of pain > 40 [15]. If we consider this threshold to be appropriate, only 1200 of the 1603 patients whose WOMAC pain and functional-capacity scores were calculated should have undergone surgery (74.9%). A breakdown of the number of inappropriate interventions (403; 25.1%) by hospital and surgery type reveals a variability, expressed as the percentage of patients with a baseline pain or functional capacity score ≤ 40 points, of 10%-35.7% for the THR group and 7.3%-41.7% for the TKR group. However, the inter-hospital variability (WCV_5-95_) was 0.24 for both surgery groups, which can be considered to be low.

Patients who underwent inappropriate knee surgery were older than those with an appropriate intervention (p < 0.001). In the appropriate surgery group for both types of procedures, men were younger than women (p < 0.05), but in the inappropriate surgery group, there was no significant difference in the mean age of men and women (p = 0.942 and p = 0.183 in TKR and THR respectively).

There were more inappropriate interventions among men than women in both types of procedures (OR 1.7 in TKR and 1.6 in THR), meaning that men were operated at lower scores on the WOMAC scale.

Nowadays, it is possible for patients to have higher levels of activity even at older ages and to need surgery earlier in order to maintain an adequate quality of life, especially men with heavy jobs that require an earlier intervention even with lower scores at WOMAC index. However, these hypotheses cannot be answered by the study design.

Since osteoarthritis is one of the most common reasons for primary care consultation in Spain, with a significant socioeconomic cost of around 511 million Euros per year [39], it seems reasonable that clinical and hospital management strategies should adopt criteria for selecting candidates and for reassessing patients on waiting lists. Simply providing more resources to regions with longer waiting lists without monitoring their surgical intervention rates may well be an inappropriate policy because the intervention is likely to be indicated for lower levels of severity [23].

The limitations of this study include the fact that clinical data collection was performed in the hospital activity environment, so the quality of some data might be affected by the typical fluctuations in activity, for example, at peak times. In relation to this, the unequal recruitment rate in the different centres should also be noted. Although we do not believe this have influenced the overall outcomes of the study, it might have affected the accuracy of the estimates in some centres. Further, it was only possible to break the samples down by centre, not by surgeon, although we assume that the criteria followed by different surgeons within the same service tend to be similar.

In relation with the study performed by Quintana et al (which was used as reference because patients' typology was very similar to ours) we assumed that its limitations (an algorithm not externally validated and a high percentage of missing values-approximately a quarter of patients who completed the baseline questionnaires did not respond to the follow-up questionnaires) did not invalidate the conclusions derived from our results. These limitations were not a major problem for authors because, on the one hand, the validity of this algorithm was supported by the similar results obtained by other research groups, and on the other hand, the high percentage of missing values did not result in statistically significant differences in relevant variables when comparing responder patients with no responders.

Finally, the study excludes private hospitals, which might provide a different picture to that obtained from the public sector.

## Conclusions

In conclusion, the type of patient undergoing primary arthroplasty appears to be similar in the different hospitals assessed, although the differences in the clinical criteria followed when indicating surgery mean that approximately a quarter of interventions performed could be considered to be inappropriate. Further research is therefore required to establish guidelines for indicating this type of surgery in order to make better use of existing resources.

## Abbreviations

TKR: (Total Knee Replacement); THR: (Total Hip Replacement); EPISER: (Study on the Prevalence of Rheumatoid Diseases in the Spanish Population); SF: (Short-Form); WOMAC: (Western Ontario and Mcmaster Universities); BMI: (Body Mass Index); SD: (Standard Deviation); 95% CI:(Confidence Interval at a 95% confidence level); EQ: _5-95 _(Extremal Quotient for the percentile range 5-95); CV: _5-95 _(Coefficient of variance for the percentile range 5-95); WCV: _5-95 _(Weighted Coefficient of Variance for the percentile range 5-95)

## Competing interests

Raquel Cobos Campos, Amaia Latorre Ramos, Felipe Esteban Aizpuru Barandiaran, Jose Ignacio Guenaga Sustaeta, Cristina Sarasqueta Eizaguirre, Antonio Escobar Martínez, Lidia García Pérez and Carmen Herrera Espiñeira declare that they have no competing interests.

## Authors' contributions

RC was in charge of reviewing the state of the scientific evidence, the draft and revision of the manuscript and the interpretation of the results. AL completed the statistical analysis and took part in the interpretation of the results. FA was involved in the design of the preliminary study, the revision of the manuscript and the interpretation of the results. AE promoted and designed the preliminary study. The remaining co-authors were involved in the recruitment of participants, data collection and quality control. All authors read and approved the final manuscript.

## Pre-publication history

The pre-publication history for this paper can be accessed here:

http://www.biomedcentral.com/1471-2474/11/249/prepub
